# A Peer and Volunteer Program for Patients and Their Families in the Pediatric Intensive Care Unit: A Pilot Program Evaluation

**DOI:** 10.3389/fped.2021.711083

**Published:** 2021-11-03

**Authors:** Nichole Pereira, Christine MacDonald, Ashley Drobot, Alexandra Bennett, Al-Bakir Ali, Daniel Garros

**Affiliations:** ^1^Pediatric Intensive Care Unit, Stollery Children's Hospital, Alberta Health Services, Edmonton, AB, Canada; ^2^Health Systems Evaluation and Evidence, Provincial Clinical Excellence, Alberta Health Services, Edmonton, AB, Canada; ^3^Department of Pediatrics, Faculty of Medicine and Dentistry, University of Alberta, Edmonton, AB, Canada

**Keywords:** patient and family centered care, peer support, volunteer, non-medical interaction, pediatric intensive care

## Abstract

**Introduction:** Patients in the pediatric intensive care unit (PICU) are at risk of developing long-term morbidities following recovery from their critical illness. One such health outcome is called post-intensive care syndrome (PICS). PICS in pediatrics may be mitigated by interventions that facilitate adjustment to the PICU setting.

**Methods:** The PICU implemented a two-pronged Peer and Volunteer (P/V) Program to help: (a) families adjust to the PICU experience with the support of a peer mentor (PM); and (b) patients receive non-medical interaction from trained volunteers (V). We designed a mixed-methods program evaluation targeting perspectives and feedback from PICU families and healthcare professionals (HCPs).

**Results:** All stakeholder groups agreed that the PICU P/V Program was a valuable resource for PICU patients and their families. HCPs reported that they lack both time and training to provide regular developmental care to patients. However, the P/V Program may influence both families' and HCP's confidence in their ability to offer non-medical interaction to children in the PICU.

**Discussion:** Important initial and on-going strengths and barriers to successful implementation were identified, including the need to clarify roles and intervention scope. The program evaluation served as a change management strategy and also helped to identify both areas for improvement and strategies for on-going sustainability. HCP's exposure to the program and modeling by PMs may have helped HCPs to feel that it is within their job description and capacity to provide emotional support and guidance to families.

## Introduction

Post-intensive care syndrome (PICS) is the new or worsening impairment in various domains of health arising after critical illness and persisting beyond acute care hospitalization (1). This syndrome is well-established in adults, but less is known about the short and long-term effects on pediatric patients following admission to pediatric intensive care units (PICU). Pediatric impairment following intensive care admission can occur in one or more of the following four domains: physical, cognitive, emotional and/or social (1). The physical domain describes the patient's functional status and/or physical disability. The cognitive domain includes attention, memory, behavior, motor coordination, inhibitor control, visual motor integration, IQ, and academic performance. The emotional (or psychological) domain includes mental health-related considerations such as irritability, avoidance, and post-traumatic stress response. The social domain exists in acknowledgment that the pediatric patient is situated within an interdependent family unit (2). While defined separately, these four domains are recognized as interconnected and interdependent (1).

In pediatrics, the following factors affect a patient's baseline health and lasting impairment in the physical, cognitive, emotional and social domains: length and type of PICU admission, the number of medical procedures performed in the PICU, cardiac arrest and/or receipt of advanced life support, invasive mechanical ventilation, receipt of sedation and opioids, and family function and structure (2). The effect of PICS on former PICU patients is variable, with several different trajectories of recovery. Though the length or degree of recovery is not predictable, PICS in pediatrics may be mitigated by interventions that facilitate adjustment to the intensive care setting and to a patient and family's “new normal” following PICU admission (3).

Developmental care is a popular concept in the field of neonatal intensive care that is known to affect neonate's physical, cognitive, emotional and social domains of health. Developmental care encompasses an approach to individualize infant's care to maximize neurological development and reduce long-term cognitive and behavioral problems (4). There is a focus on intentionally working to meet developmental milestones by clustering care to allow sleep, bonding, brain stimulus, growth, coordination, etc. (4–6). Developmental care has traditionally received less attention in PICU settings, although interventions to facilitate developmental care in pediatrics is growing.

Patient and Family Centered Care (PFCC) describes both a philosophy and practice in pediatric healthcare (7). The philosophy acknowledges that healthcare should be a collaborative effort based on patient, family, and healthcare provider (HCP) mutual understanding of reality, and based on mutual trust and respect for the other's perspectives, opinions, and goals (8, 9). In practice, PFCC looks like open and honest communication, shared prioritization, and an on-going partnership to reach defined goals (8, 9). Since 2008, the Stollery Children's Hospital in Edmonton, Canada has committed to the integration of PFCC into hospital philosophy and practice (10). Local organizational improvement initiatives are both generated through, and vetted by, the PFCC council and its members.

The aim of this program evaluation was to facilitate PFCC at the Stollery Children's Hospital by implementing and evaluating a two-pronged service to assist with patient and family experience following admission to the PICU; a Peer Mentor and Volunteer (P/V) Program for patients and their families. Specifically, our goals were to: (a) offer patients age-appropriate, non-medical interaction *via* volunteers (V), and (b) offer families guidance through their hospital experience *via* peer mentors (PM).

## Methods

We started this initiative by establishing a multi-disciplinary team which consisted of Child Life Specialists, the Stollery Volunteer Coordinator, PFCC Advisors, Social Workers, a PICU Clinical Nurse Educator, a Clinical Nurse Specialist, and a Physician to lead this initiative and to recruit and train Vs and PMs. Child Life Specialists are trained professionals in Canada who assist children and adolescents to cope with the impact of hospitalization, by assessing, planning, implementing, and evaluating mutually agreed upon goals. They are well positioned to engage with both patients and their families about social, psychological, and developmental-related needs while in hospital.

We defined Vs as those trained to provide age-appropriate, positive and non-medical interaction to PICU patients, including cuddling, rocking, reading, playing, coloring, singing, crafting, or hand holding. To be eligible, Vs needed to be >18 years of age and have had inpatient Stollery V experience. This service was offered by Vs during the day to patients in the PICU. The V portion of the program was intended to support patients especially when their families were taking self-care breaks or were busy with other life demands such as work or family.

We defined PMs as those with a shared understanding of the PICU family experience from having previously had their child admitted into the PICU. PMs were positioned to provide peer support, normalization, validation, and guidance throughout current PICU families' hospital stays. PMs were available during the day and in the evenings. PMs and Vs were recruited *via* the Stollery Children Hospital's normal volunteer intake program. If they met the criteria as defined above, they were connected to the PICU P/V project team for further information and training. Training for both positions included on-line and in-person modules about the organization and hospital, patient, and family centered care goals; effective communication; confidentiality; professional boundaries; self-care; and how to facilitate appropriate referrals. The team also partnered with Stollery Social Workers to develop a new 2-h cultural sensitivity course, and Vs and PMs were encouraged to attend. PMs and Vs were required to have passed a security clearance check prior to training. Following training, Vs were supported by Child Life Specialists who helped to coordinate appropriate patients to engage with and direct the activity of that engagement. PFCC Advisors took an active role in supporting PMs, and helped to direct them to appropriate families. Families could also request Vs and PMs *via* PICU HCPs or directly *via* phone or email to the PFCC department.

### Procedures and Protocols

While the primary outcome measures for this evaluation were related to patient and family experience and satisfaction, we recognized that change management with the PICU multidisciplinary team was an important factor for this program's success and sustainability. Thus, we designed a mixed-methods program evaluation with the help of our hospital's Department for Health Systems Evaluation and Evidence. We received ethics approval for this program evaluation from the University Of Alberta Research Ethics Board (Study ID Pro00089760). Data were collected through the course of the program's two phases of implementation in September to November, 2019 (pre-implementation) to January to March, 2020 (post-implementation). The evaluation commenced with a HCP focus group. Surveys were then distributed at pre- and post-program implementation. Surveys were developed for PICU HCPs and Family/Caregivers (henceforth “family/caregiver” is referred to as just “family”). All surveys and the focus group guide were developed collaboratively by the PICU and evaluation teams. All family data were collected prospectively while families were present in the PICU and their child was an admitted patient.

#### PICU HCPs Focus Group

A 1-hour focus group discussion took place prior to program implementation as a change management strategy to gather HCPs feedback and buy-in on: (a) what would enable the successful launch of the program, (b) their expectations of the program, and (c) the perceived benefits and challenges to implementing such a program. The evaluation team conducted the in-person focus group at the hospital with voluntary participants. The PICU team supported focus group recruitment by email until there was HCPs representation from each of the major stakeholder groups.

#### PICU HCPs Survey

The PICU HCPs Survey targeted PICU Nurses, Physicians, Child Life Specialists, Unit Clerks, Health Care Aids, Allied Health (i.e., Respiratory Therapists, Social Workers, etc.), and Patient and Family Centered Care. The evaluation team was responsible for both pre- and post-implementation survey distribution. All HCPs were invited to complete the pre-survey. The post-survey was also sent to all HCPs, though the questions were directed to those who had exposure to the PM or V Program.

#### Volunteer and Peer Mentor Surveys

The V Survey targeted PICU families for their perspective on receiving support from a PICU V. The PM Survey targeted PICU families for their perspective on receiving support from a PICU PM. Both surveys were offered to a convenience sample of families in the PICU at program pre-implementation. Families were asked to participate in the pre-survey if their child was in the unit for at least 3 days so that they were familiar with the team, environment, and routine. Families were only offered surveys if their child was “stable,” meaning they were not actively decompensating or deteriorating or experiencing increased acuity. We also avoided families whose child was at end of life, or if it was a critical decision-making day. Surveys were offered when the PICU team was available to offer surveys (Monday to Friday, 8:00am−4:00pm). Families were asked to participate in the post-survey at the same time of day as pre-implementation, and only if they had exposure to the PM and/or V Program.

The V and PM Surveys were distributed both by paper and online through the *SelectSurvey* platform. The PICU team handed out paper surveys and iPads on the unit. Completed paper surveys were dropped in a locked survey box on the unit. Only the evaluation team had access to the locked survey box to retrieve and enter data from the paper-based surveys. Table 1 shows the potential data limitations and corresponding activities and strategies used to limit bias throughout this evaluation.

**Table 1:** Data limitation activities and strategies.

**Data collection activity**	**Strategies taken to avoid bias**
**HCPs survey**	• Made the survey available in an online format • Sent three reminder emails to increase the response rate, thereby moving the invitation to participate at the top of HCPs inboxes • Extended the data collection period
**HCPs focus group**	• Sent three invites to PICU HCPs to ensure a higher rate of responses
**V survey**	• Received input from the Child Life and Patient and Family Centered Care Teams in the design of both surveys • Made the surveys available in both online and paper format
**PM survey**	• Extended the data collection period until the program was suspended (pandemic)

### Analysis

Analysis was conducted using Microsoft Excel to theme qualitative survey comments and to generate descriptive statistics and visualizations of quantitative data.

## Results

### Pre-program Implementation Results

#### HCPs Focus Group

Five HCPs voluntarily attended the scheduled focus group. Participants included nurses, occupational therapy, and Volunteer Coordinator who provided their perspective on expected program benefits, program communication, and program operations. Key themes that emerged about expected program benefits centered on facilitating the families' transition into the PICU environment and providing non-medical support, which was expected to help the families as well as HCPs who may see a decrease in non-medical or developmental care responsibilities.

Participants suggested key communication requirements, like how to disseminate information and clarify roles and responsibilities. Participants identified poor communication as a potential barrier to program rollout, and shared suggestions for program operations including sharing communications through the management team. They also recommended resources for Vs, and opportunities for HCPs to build rapport with and provide feedback to Vs. Participants identified that it would be important for HCPs to keep an open mind about the Vs, and remember that they would be there to help ease HCPs workload. The focus group themes are summarized in Table 2.

**Table 2:** HCPs focus group themes (pre-implementation).

**Theme**	**Focus group feedback**
**Expected Program Benefits for Families**	The P/V Program is expected to support families through: • Non-medical support • Someone who they can share their experience with • Talking about their child in terms other than their medical condition • Alleviating family worries and anxieties about child's care • Helping families be unguarded around HCPs • Clarifying families' roles and expectations • Providing a break from the bedside • Interacting with other children in the family to give families time to spend with the child receiving care
**Expected Program Benefits for HCPs**	The P/V Program is expected to support HCPs through: • Offloading non-medical interactions to the Vs • Freeing HCPs capacity in other areas with Vs supporting developmental care • Encouraging a collaborative experience between providers and Vs
**Program communication—approaches for HCPs**	Suggestions for successful program adoption: • Early engagement about the program purpose • Using multiple communication approaches to raise awareness • Online communication about program (email or Lunch and Learn sessions) • In-person communication about program (during charge nurse rounds, education rounds with a ‘champion', or in ‘pod talks') • Communication from ‘unit champions; to help promote the program and engage others • Display board in common areas of the unit • Open communication about program performance
**Program communication—information for HCPs**	• Collaboration with HCPs (nurses and allied health teams) to clarify their roles and the Vs' roles • Specific details on the Vs' role: ° How the V could work alongside the nurses and social work team ° V training outline ° V reporting structure ° V confidentiality ° How boundaries with families would be maintained ° Processes for V to report child safety issues ° What supports would be in place for Vs to handle triggers • When Vs would be on unit • How to request Vs (ex: a referral form)
**Program communication—approaches for new families**	• Welcome packages for new families to build program awareness • Advertising program *via* family coffee hour
**Program rollout—potential barriers**	• Lack of communication and not having early communication of program information
**Program operations—**	• Involving management from the beginning
**management team**	• Management share outcomes of the program rollout as it progresses to increase buy-in from HCPs
**Program operations—V resources**	• Formal on-unit introductions of Vs to HCPs by V supervisor, coordinator, or Child Life • Process maps to show Vs what spaces they can use • Mini-checklists that tell Vs what to do on their shifts
**Program operations—HCPs/V relationship**	• Activity log detailing if Vs have visited with a patient to keep nursing HCPs informed about V activities • HCPs expectation that program Vs and nurses need to collaborate and check-in with one another about appropriate times to interact with a patient • Supporting rapport between Vs and nurses • Opportunity for HCPs to provide direct feedback to Vs • Ensure HCPs maintain an open mind toward the Vs and understanding Vs are intended to help HCPs

#### HCP Pre-implementation Volunteer and Peer Mentor Surveys

**Figure 1:**
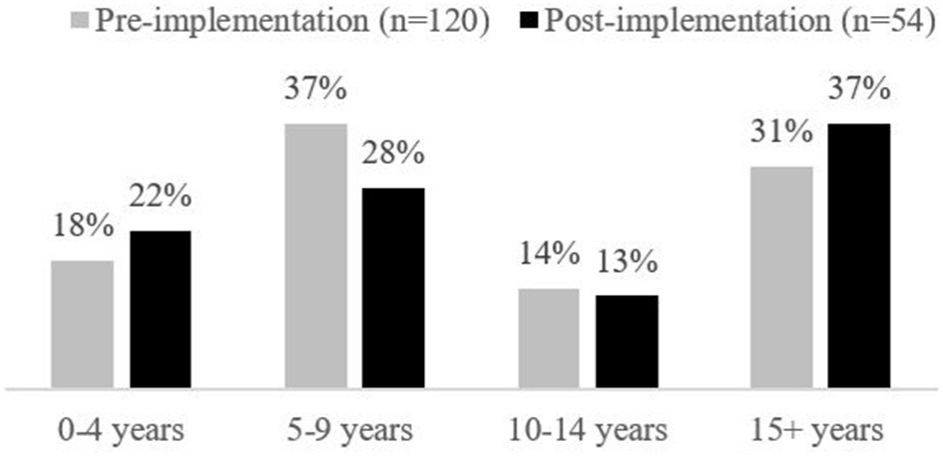
Staff years of experience in healthcare.

**Figure 2:**
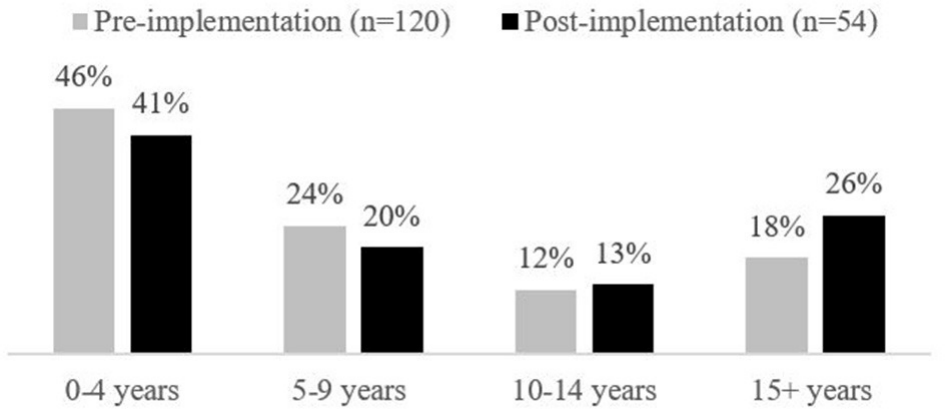
Staff years of experience in pediatric intensive care setting.

A total of 120 PICU HCPs responded to the Volunteer and Peer Mentor pre-implementation survey (response rate of 34% or 120/351 HCPs; see Figures 1, 2 for the demographic breakdown of HCPs who responded). Over half of the respondents were nurses (65%, *n* = 78), followed by allied health (19%, *n* = 23), and physicians (8%, *n* = 9). Of those respondents who identified their discipline as allied health, the majority were respiratory therapists (12%, *n* = 14). The disciplines of the remaining respondents included: unit clerks, social workers, health care aides, a PICU administrator, occupational therapists, and a pharmacist.

##### HCP Survey on the Volunteer Program

**Figure 3:**
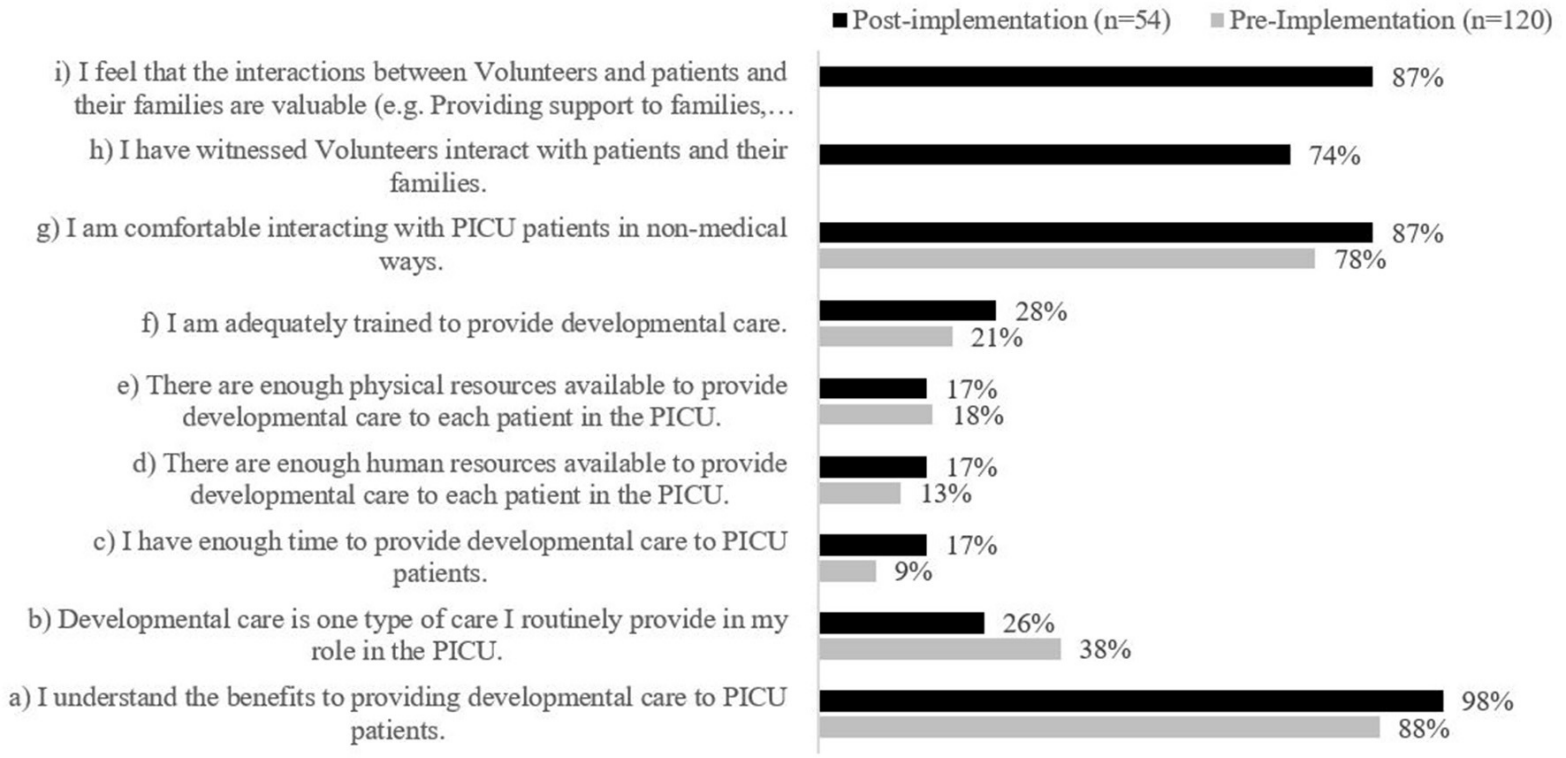
Staff level of agreement regarding statements on developmental care and the volunteer program pre- and post-implementation. Statements (h) and (i) were not included as survey items in the staff pre-implementation survey; therefore, no data is displayed.

Prior to program implementation, HCPs reported they understood the benefits to providing developmental care to PICU patients (88%, *n* = 105) and that they were comfortable interacting with PICU patients in non-medical ways (78%, *n* = 94). However, HCPs responded that there were not enough resources available to provide developmental care to PICU patients, and only 38% (*n* = 45) reported providing routine developmental care (Figure 3).

**Figure 4:**
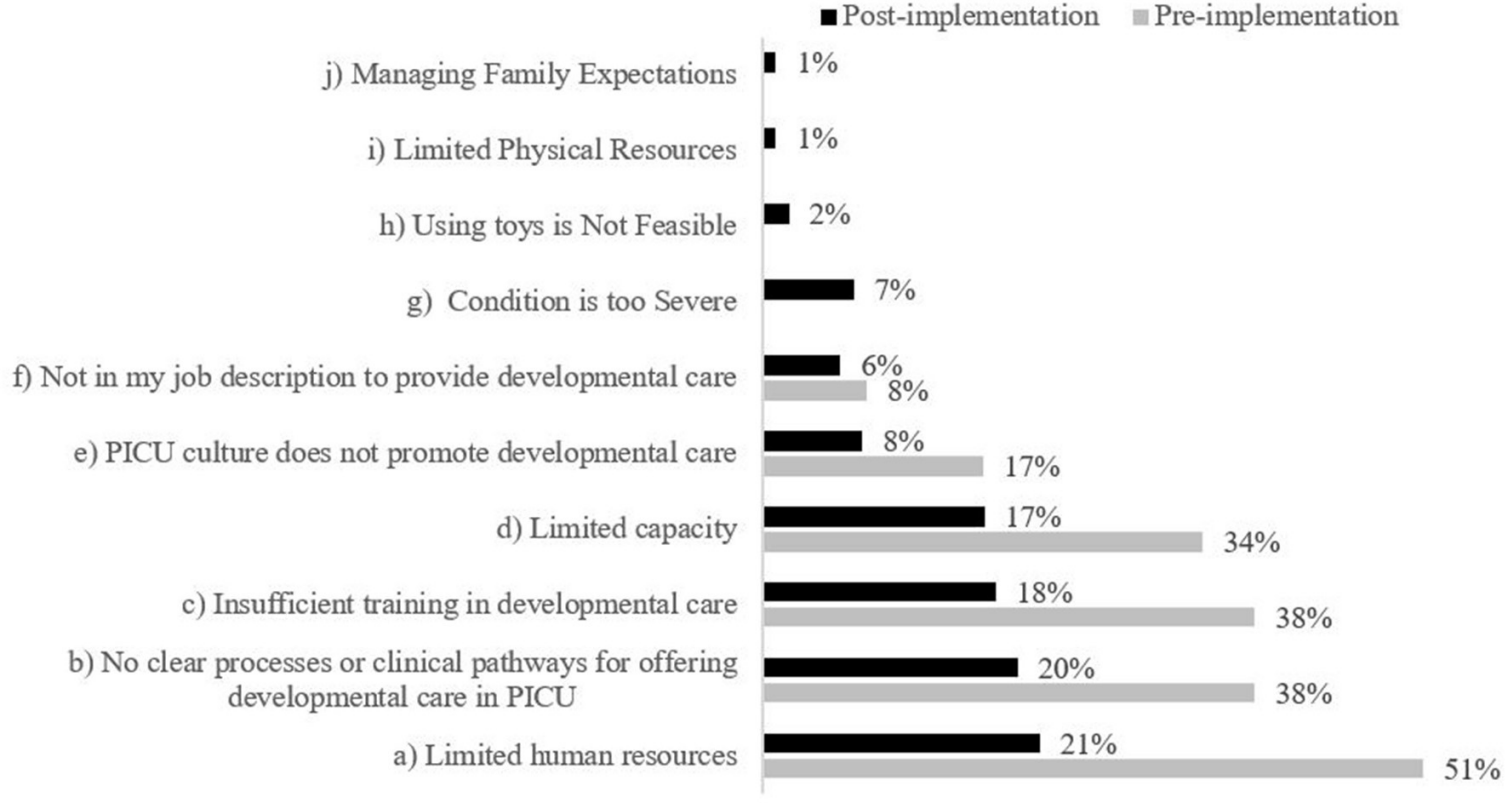
Staff identified barriers to providing developmental care in PICU. Respondents could select multiple responses; therefore, n will not total 120 (pre) and 54 (post). Statements (g)–(j) were not included as survey items in the staff pre-implementation survey; therefore, no data is displayed.

Most HCPs agreed that there were barriers to providing developmental care in the PICU (74%, *n* = 89). HCPs further elaborated that their ability to provide developmental care was dependent on their daily duties and how busy it was on the unit, and most frequently identified limited human resources (51%, *n* = 61), insufficient training (38%, *n* = 46), and no clear processes or clinical pathways for offering developmental care in PICU (38%, *n* = 45) as barriers (Figure 4). While HCPs reported that some developmental care was provided in the PICU, they felt that the V Program could further enhance developmental care and would be valuable to help address limited HCPs capacity.

When asked how likely they were to recommend a potential V Program to families on a scale of 1–10, where 10 is “extremely likely,” HCPs indicated a mean rating of 7.63. When asked *via* open text response, some HCPs felt that the V Program would improve access to regular development care and ease families' worries when they cannot be present or need a break. HCPs felt such a program would be most useful for low level acute care patients who could benefit most from the interaction. Some HCPs cautioned that the program may not be beneficial to all patients in the PICU, particularly those who are acutely ill as they are often sedated, intubated, and cannot respond to play activities. HCPs felt that some families may not want strangers to touch their child and that untrained Vs could interfere with HCPs providing medical care. HCPs also emphasized the potential role of the V to educate and serve as patient advocates to families, in that they provide compassion and understanding to patients, suggesting Vs might also build families' confidence and decrease their anxiety about interacting with their child.

##### HCP Survey on the Peer Mentor Program

**Figure 5:**
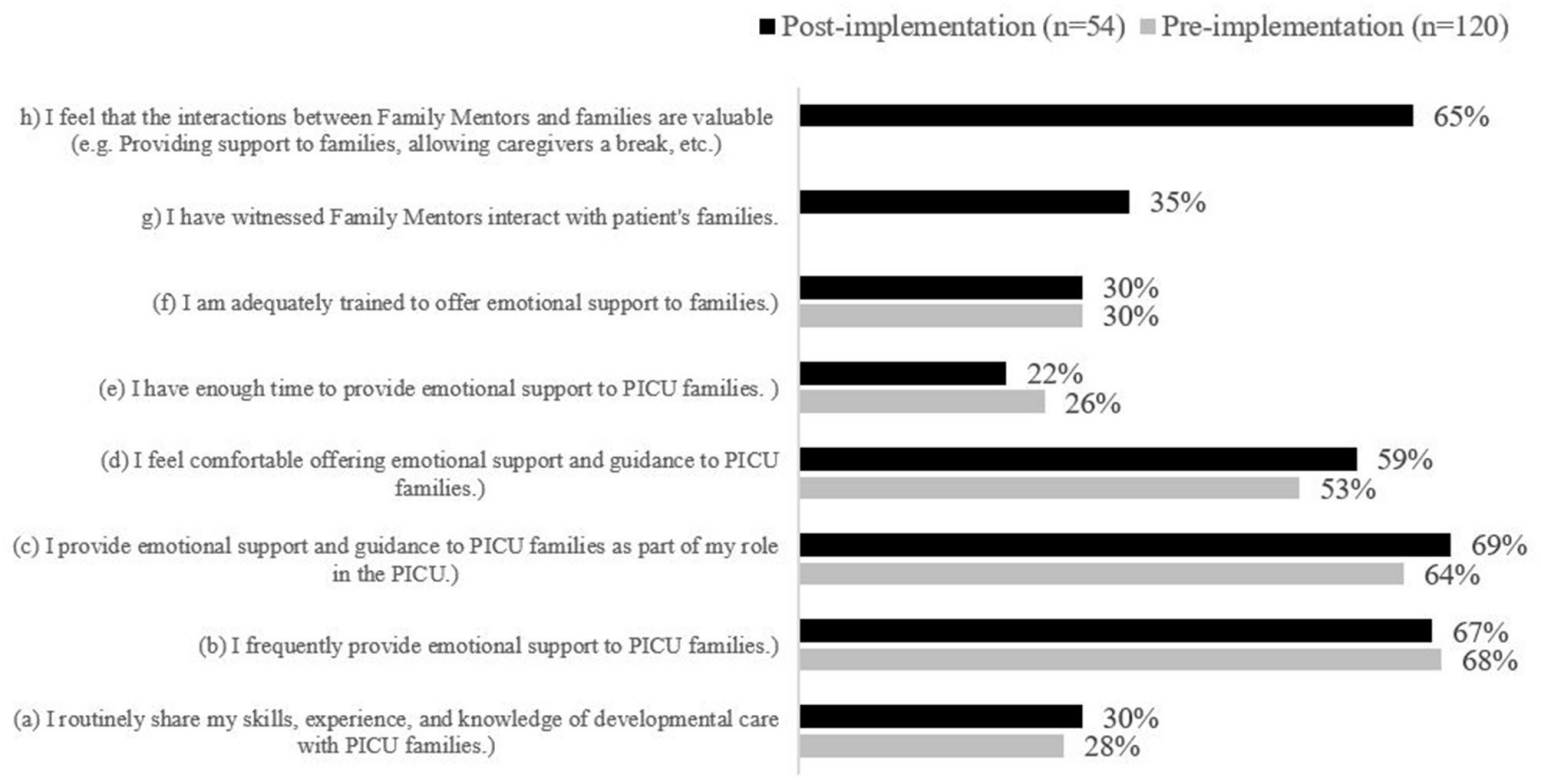
Staff Level of Agreement with Statements regarding the Family Mentor Program. Statements (g) and (h) were not included as survey items in the staff pre-implementation survey; therefore, no data is displayed.

**Figure 6:**
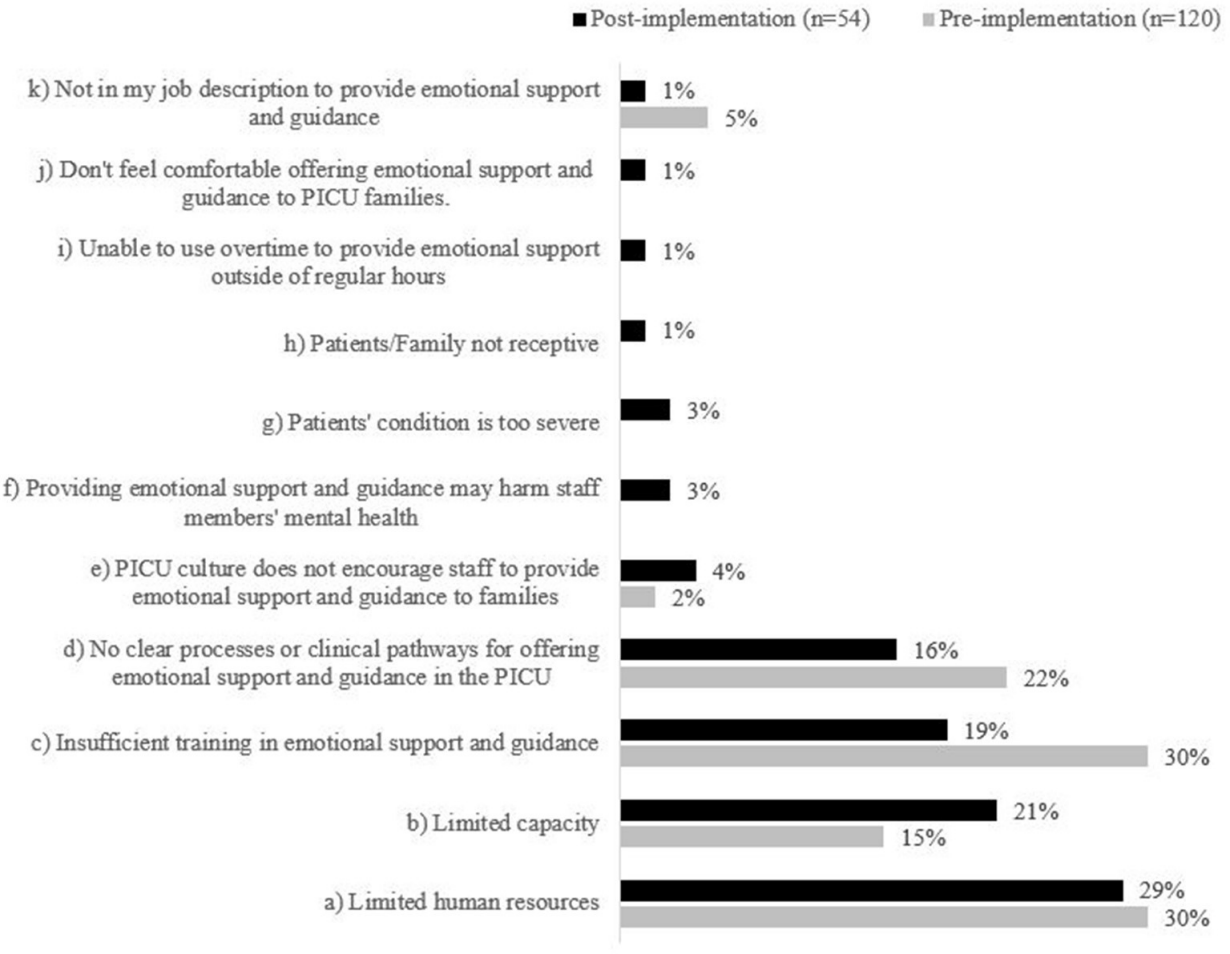
Staff reported barriers to providing families with emotional support & guidance. Items (f)–(j) were included in the post-implementation survey only.

Most HCPs reported that they frequently provided emotional support (68%, *n* = 82) and guidance to families in the PICU (64%, *n* = 77) and were comfortable doing so (53%, *n* = 64), but far fewer HCPs agreed that they routinely shared their skills, experience, and knowledge of developmental care with PICU families (28%, *n* = 34). Moreover, few HCPs agreed that they had enough time or adequate training to provide emotional support to PICU families (26%, *n* = 31; and 30%, *n* = 36, respectively; Figure 5). Fifty percent of HCPs reported barriers to providing emotional support and guidance in the PICU (*n* = 60), while 34% (*n* = 41) reported no barriers. The barriers HCPs most frequently identified were limited human resources (30%, *n* = 36), insufficient training (30%, *n* = 36), and a lack of clear processes for offering emotional supports (Figure 6).

When asked how likely they were to recommend the PM Program to families on a scale of 1–10, where 10 is “extremely likely,” HCPs indicated a mean rating of 7.63. When asked to freely comment on this, some HCPs felt that the program would be an asset to families and long overdue in the PICU, expressing excitement for its launch. HCPs elaborated that the program may be helpful in providing support to families at a peer-to-peer level rather than *via* the medical team. HCPs recommended specific training to promote the success of the program including, ensuring that mentors be trained with a clearly identified scope of practice to avoid overlap with other professionals, in particular social work. HCPs further recommended that training should include how to select appropriate patients to engage based on patient status, guidelines on developmental care, and training to avoid post-traumatic stress.

#### Family Pre-implementation Volunteer Program Survey

A total of 25 families responded to the V Program pre-implementation survey.

**Figure 7:**
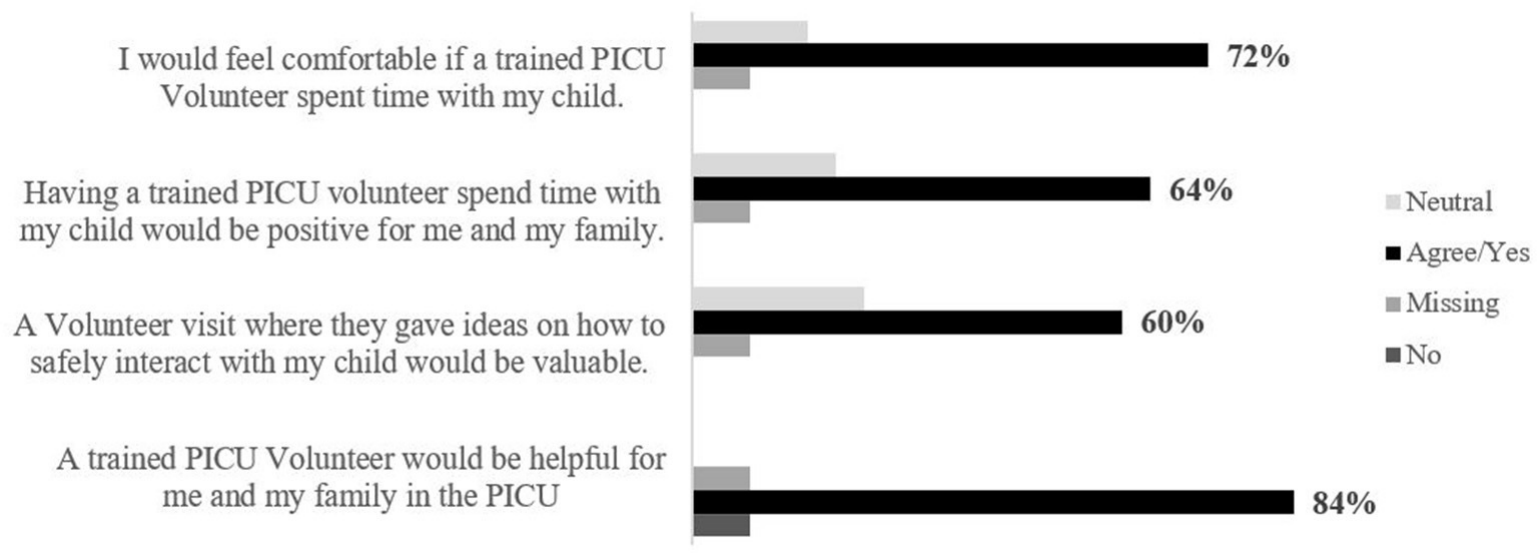
Parent level of agreement on volunteer program benefits (pre-survey).

Overall, families felt comfortable in the position of having a trained PICU V spend time with their child (72%, *n* = 18), and 84% (*n* = 21) of respondents further agreed that access to a trained V would be helpful for them and their family (Figure 7). Families felt the Vs would help by: (a) allowing their children to focus on something other than illness; (b) providing families a break to reconnect, turn off emotional stress, go home and shower; (c) allowing for their child to learn how to play; (d) helping overcome monotony and enrich further socialization; and (e) allowing other adults to interact with the infant when the family cannot be present due to emotional stress and/or the family living far from the hospital. Families expressed interest in receiving ideas from a V in how to interact with their child, in alignment with developmental care best practices (60%, *n* = 15). Families also agreed that play and interaction from a PICU V would make a difference in their child's recovery (56%, *n* = 14).

**Figure 8:**
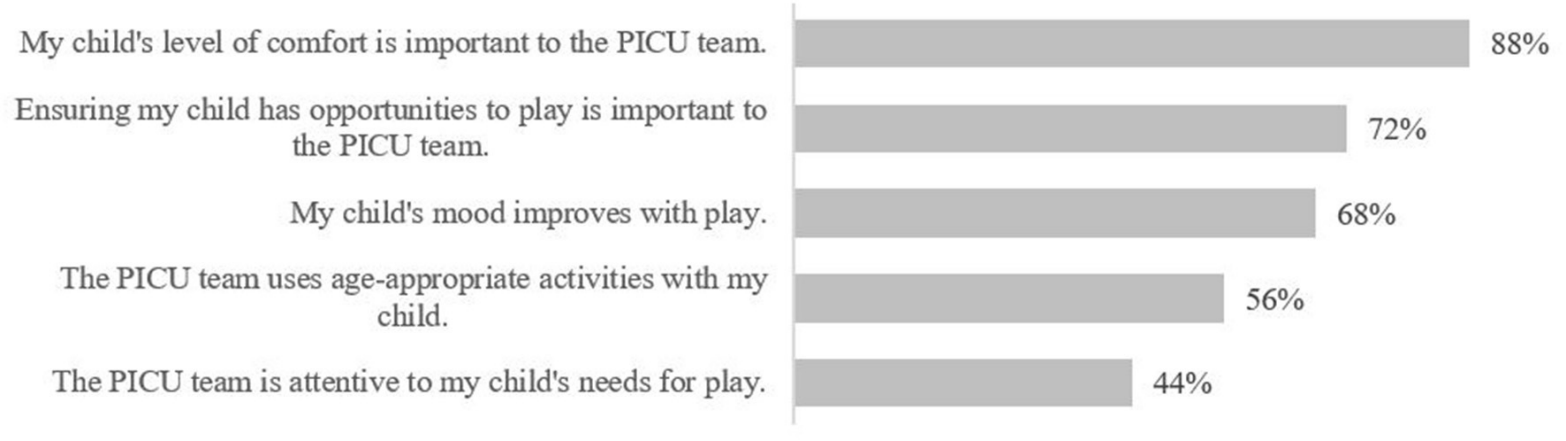
Family level of agreement on PICU experience statements (volunteer, pre-survey).

Most families felt positive about their experience in the PICU; however, families felt least positive about the level of playing and developmental care their child received in the PICU (see Figure 8 for survey item breakdown). Most families disagreed that there were barriers that prevented them from interacting with their child in the PICU (68%, *n* = 17). Families reported exhaustion (*n* = 2) and fear of touching/dislodging tubes (*n* = 1) as barriers to interacting with their child. Four families did not reply to the survey item.

#### Family Pre-implementation Peer Mentor Program Survey

**Figure 9:**
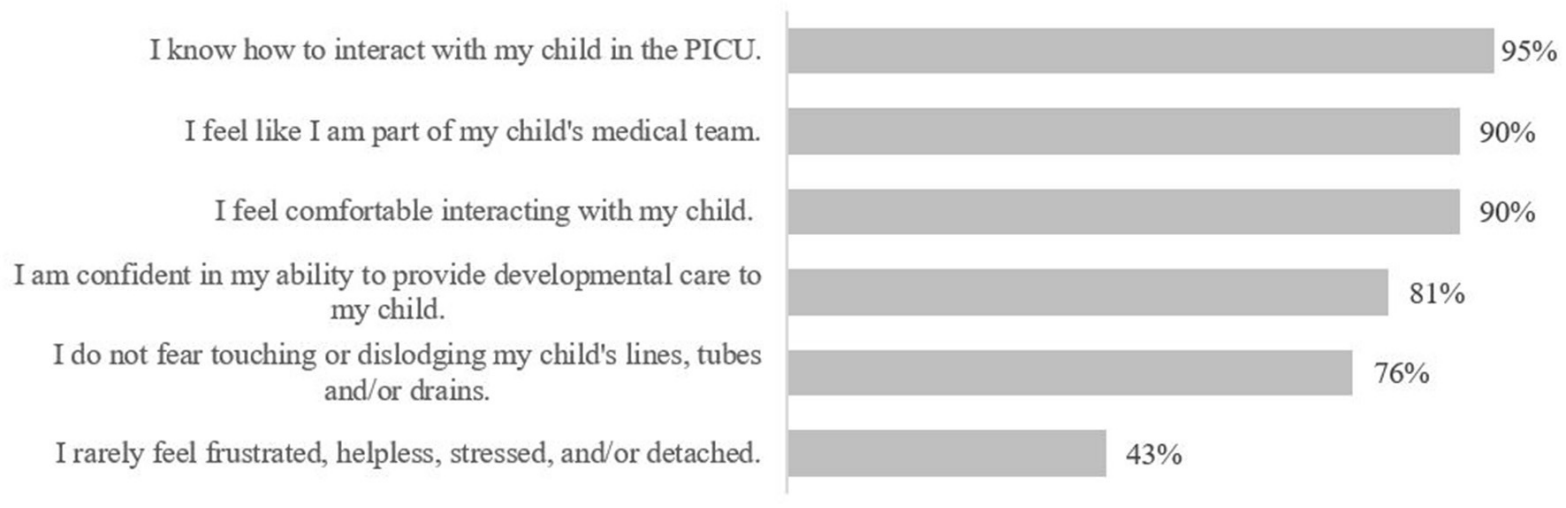
Family level of agreement in PICU experience statements (peer mentor, pre-survey).

**Figure 10:**
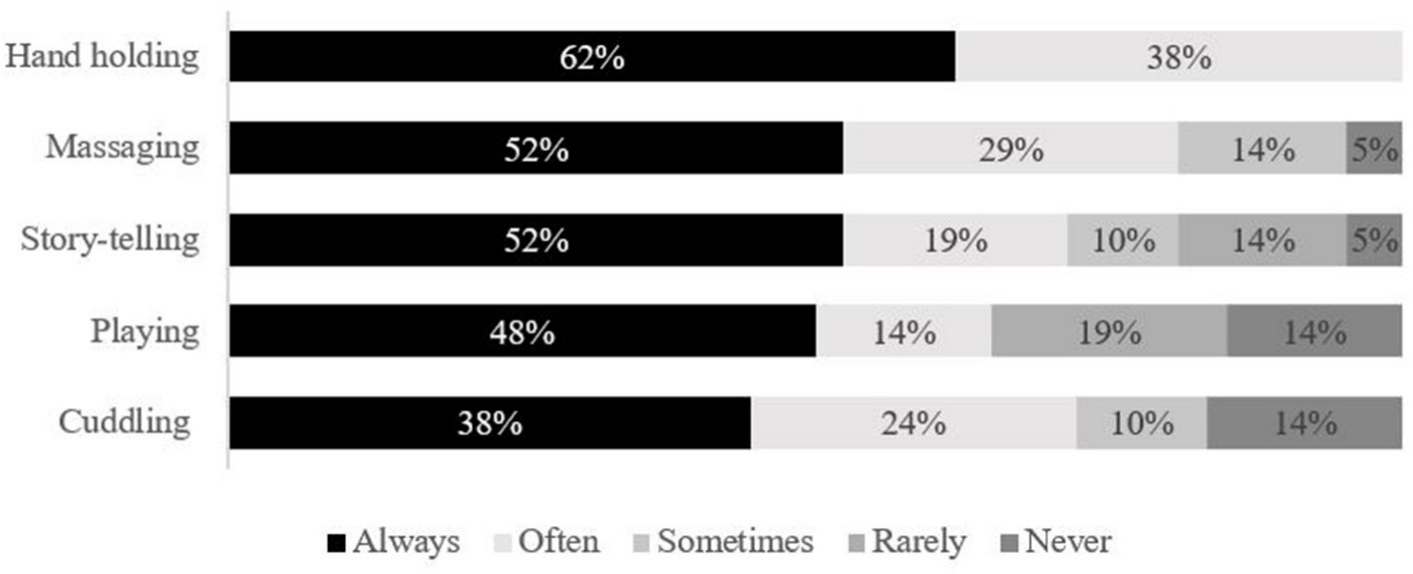
Family primary interaction with child in PICU (peer mentor, pre-survey).

A total of 21 families responded to the PM survey; just over half were admitted to the PICU for their first critical care experience (52%, *n* = 11). On average, seven families reported spending an average of 88 h per week with their child in PICU (mean: 70 h; range 8–168 h). Fourteen families skipped this question. Most families felt comfortable with their understanding of how to interact with their child and HCPs in the PICU but reported commonly experiencing negative feelings and emotions (Figure 9). Families were asked about how frequently they engaged in a variety of activities with their child in the PICU; hand holding was the most commonly reported activity, followed by massaging and story-telling, while playing and cuddling were reportedly engaged in least (Figure 10). Families referenced being exhausted, uncomfortable seating at the bedside, and machine supports that prevented cuddling/holding (e.g., intubation) as barriers to interacting with their child. One respondent did not answer the survey item.

**Figure 11:**
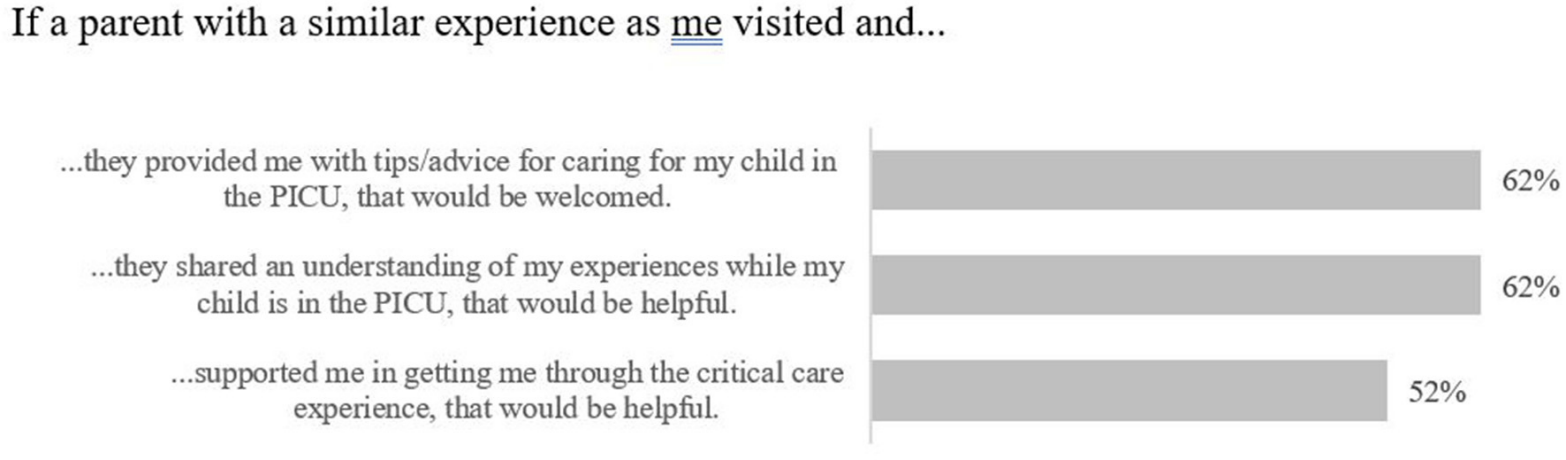
Family level of agreement in PICU experience statements (peer mentor, pre-survey).

When questioned about their perspectives on a potential PICU PM program, most families indicated they would welcome support from families who had similar PICU experiences and felt such support would be helpful (Figure 11). Just over half of families indicated they would feel supported by a PM (52%, *n* = 11); however, some families reported that their interest would depend on the mentor's personality. Families reported they would seek advice from a PM about the treatment experience, coping strategies, advocating for their involvement in the care team, and balancing life in the PICU (see Table 3). Most families felt that there was a difference between speaking to someone who has had a similar experience as them compared to speaking to a health professional (57%, *n* = 12). They perceived that health professionals would provide clinical expertise and a neutral understanding of clinical circumstances in the PICU, and that PMs would provide personal, open, and emotional support that would make them feel as if they were not alone. Families expressed that PMs may potentially have biases and different values, and they identified that they may lack familiarity with each case.

**Table 3:** Areas of advice families desired from PMs (pre-survey).

**Theme**	**Example**
**Treatment**	• How was treatment? • Did the PICU have answers for you throughout the process? • Was there a plan set in motion?
**Coping**	• How do you provide your own self-care? • What coping mechanisms did you use especially during long visits? • What do you do when your child is in pain or discomfort?
**Advocacy**	• Do families get a say/opinion on the process or the treatment? • How did you advocate for your child?
**Life Balance**	• How do you balance everything? (e.g., family, home, hospital, etc.) • What do I bring when staying the night?

### Post-program Implementation Results

The post-program evaluation surveys were distributed starting in January 2020. PICU team HCPs approached families who were known to have received either PM and/or V support in the PICU. As families are not present in the PICU all of the time, it was difficult to connect with families to obtain their feedback on the services they and their child received. Unfortunately, in March 2020, this program evaluations was forced to suspend due to the COVID-19 pandemic hospital restrictions on families and Vs allowed in the hospital. Additionally, at this time PICU HCPs were re-deployed to train in other areas of the hospital. Thus, the response rate of the HCPs and family P/V post-program evaluations were affected, and we received significantly smaller response rates. The following results should be interpreted with caution.

#### HCPs Post-implementation Surveys

Fifty-four HCPs responded to the post-implementation survey (response rate of 15% or 54/351 HCPs). Overall respondents were somewhat more experienced in health care but had similar PICU experience compared to pre-implementation survey respondents (see Figures 1, 2 for the demographic breakdown of HCPs who responded and their experience in PICU).

##### HCP Survey on the Volunteer Program

HCPs reported they understood the benefits of providing developmental care to PICU patients (98%, *n* = 53) and that they were comfortable interacting with PICU patients in non-medical ways (87%, *n* = 47); however, HCPs generally did not agree that the PICU had enough physical, human, or time resources available to provide developmental care to PICU patients, and 26% (*n* = 14) reported routinely providing developmental care (Figure 3). 80% (*n* = 43) of HCPs agreed that there are barriers to providing developmental care in the PICU. As in the pre-implementation survey, HCPs most frequently identified limited human resources (21%, *n* = 25), no clear processes or clinical pathways for offering developmental care in PICU (20%, *n* = 23), and insufficient training (18%, *n* = 21), as barriers to providing developmental care (Figure 4). Post-implementation, there was a decrease in the reported presence of barriers to developmental care and HCPs indicated that the V program provides supplementary support to the existing developmental care offered in the PICU.

HCPs were asked about interactions between Vs and patients and their families. A large majority of HCPs reported witnessing Vs interact with patients and their families on the unit (74%, *n* = 40) and felt that those interactions were valuable (87%, *n* = 47). When asked how likely they were to recommend the V Program to families on a scale of 1–10, where 10 is “extremely likely,” HCPs indicated a mean rating of 8.10, an increase from the pre-survey. HCPs commented that the V's value was in providing increased access to developmental care, especially from someone who does not also provide medical care. Further, HCPs noted that that the V Program provided value in supporting families and decreasing clinicians' workloads.

##### HCP Survey on the Peer Mentor Program

Most HCPs reported that they frequently provided emotional support (67%, *n* = 36) and guidance to families in the PICU (69%, *n* = 37) and were comfortable doing so (59%, *n* = 32), but few HCPs agreed that they routinely shared their knowledge of developmental care with PICU families (30%, *n* = 16).

Moreover, few HCPs agreed that they had enough time or adequate training to provide emotional support to PICU families (22%, *n* = 12; and 30%, *n* = 16, respectively) (Figure 5). Most HCPs reported barriers to providing emotional support and guidance in the PICU (80%, *n* = 43); while 19% (*n* = 10) of HCPs felt there were no barriers. One individual did not respond to this survey item. The perceived barriers to providing families with emotional support and guidance decreased across most dimensions, except capacity. Post implementation, 21% of HCPs felt limited capacity was a barrier compared to 15% pre-implementation (*n* = 15 and *n* = 18, respectively; Figure 6). Based on conversations with HCPs, additional options were included in the post-implementation survey to select as barriers to providing emotional support and guidance; however, few HCPs identified these items as barriers. HCPs most frequently identified limited human resources (29%, *n* = 20), limited capacity (21%, *n* = 15), and insufficient training (19%, *n* = 13) as barriers to providing emotional support and guidance (Figure 6).

Only 35% (*n* = 19) of HCPs reported witnessing PMs interact with PICU patients and their families. This trend may be influenced by the post-implementation program duration being cut short due to the COVID-19 pandemic. However, the majority of HCPs reported that the interactions between PMs and families were valuable (65%, *n* = 35). When asked how likely they were to recommend the PM Program to families on a scale of 1–10, where 10 is “extremely likely,” HCPs indicated a mean rating of 7.94. HCPs felt that PMs provided peer-to-peer supports, acted as non-medical confidants, addressed family health (providing emotional support), and empowered families to be more proactive in their child's care.

#### Family Post-implementation Volunteer Survey

Over the course of 5 months, Vs visited 30 patients and offered age-appropriate, non-medical interaction to them a total of 86 times. Patients ranged in age from 4 months to 15 years old. Interactions included playing, reading, singing, cuddling, hand holding, and comforting. Interactions also included families if and when they were present and receptive to it. Twenty-six times, a V visited a patient and/or family and no age-appropriate, non-medical interaction was offered or given, either due to patient or family situation or refusal.

The PICU team approached families whose child received a V intervention for their perspective and feedback to inform the V program evaluation. The PICU team was able to connect with five families who received V support for their child. We were not able to offer all 30 families the opportunity to provide feedback, as some patient admissions were short and families were not always present in the PICU during office hours when our team was available to offer a survey. Families were also not always present to experience the V support offered to their child while they were away, and so could not offer their feedback on an intervention they had not witnessed. Two families responded to all questions, while three gave partial responses. Most respondents' children had been in the PICU for <2 weeks and had been visited by a V one to three times in the past seven days. One individual noted that interactions with the Vs were rare and had not occurred at all in the past 7 days.

All families agreed that their child's comfort was important to the PICU team. As in pre-implementation, families felt least positive about the level of playing and developmental care their children received in the PICU; however, families generally agreed that the PICU team uses age-appropriate activities with their children and are attentive to their need for play; an increase compared to the pre-implementation results (*n* = 3, 75%).

When asked about the V program, families mostly agreed that they felt comfortable with trained PICU Vs spending time with their children. Families reported that they did not agree that a V offered ideas on how to interact with their child in the PICU. Families felt neutral to positive about if having a trained V spend time with their children was positive for their families, though none disagreed. Those who felt neutral about the benefits of a V's support also felt neutral about if interaction from the PICU V made a difference in their children's recoveries, while those who agreed the Vs were positive for their families also agreed that their children's recoveries were positively influenced by Vs. One respondent was generally not in alignment with the majority of the respondents and reported being uncertain of the PICU team's attentiveness to their child's needs for play, unsure if they were comfortable with the V, and disagreed that the V offered ideas on how to interact with their child. Unfortunately, this individual did not provide explanation of any particulars that may have influenced their experiences.

Families reported PICU Vs engaged with their children through play with toys, hand holding, reading, singing, cuddling, and talking—including asking age-appropriate questions. The majority of families did not feel there were barriers preventing them from interacting with their children in the PICU (*n* = 3, 75%). One respondent cited challenges with coping, exhaustion, fear of tubing, and limited knowledge of how to interact with their child.

#### Family Post-implementation Peer Mentor Survey

Six families both received PM support and were approached and agreed to offer their feedback on the PM program. As with the pre-surveys, we employed convenience sampling, so did not approach all families for evaluation due to our inability to find a time they were present on the unit to offer feedback. Two families answered all questions and four gave partial responses. One response was excluded as the respondent gave multiple contradictory answers, which could not be reliably interpreted.

One respondent's child had been in the PICU for <2 weeks, two had been admitted for more than 3 months, and the remaining had been admitted between 2 weeks and 3 months. All families reported they had been visited by a PM one to three times in the past seven days. A third of the families (*n* = 2, 33%; no response = 3, 50%) reported the average number of hours they spend in the PICU per week, which ranged from 14 to 50 h.

Most families felt comfortable with their understanding of how to interact with their child and HCPs in the PICU, but more often reported experiencing negative feelings and emotions. All families reported they or their family members interacted with their child in the PICU in some manner, including hand-holding. Most reported engaging in story-telling (*n* = 6, 100%), massage (*n* = 5, 83%), or play (*n* = 5, 83%), but a few reported never engaging in these activities (massage *n* = 1, 17%; play *n* = 2, 33%). Overall, engaging in cuddling was reported least frequently (*n* = 3, 50%). 83% (*n* = 5) of families did not feel there were any barriers that prevented them from interacting with their child in the PICU; however, those barriers that were highlighted included difficulty coping, exhaustion, fear of touching or dislodging tubes, and physical disability (*n* = 1, 17%).

When asked about their interactions with the PM program, most families (*n* = 4, 67%) agreed that a PM provided them support that helped them through their critical care experience, but were mostly neutral about if PMs provided tips and advice (*n* = 3, 50%). Families reported talking about day-to-day life and their children (including those not in the PICU) with the PMs. Overall, families reported that speaking with a PM was helpful compared to a health professional, because they felt less alone and as if someone truly understood their experiences.

## Discussion

This systematic evaluation served as both a program evaluation for families and HCPs as important stakeholder groups, as well as a change management strategy for HCPs in the PICU. At pre- and post-evaluation, all stakeholder groups mostly agreed that the PICU P/V Program was a valuable resource for PICU patients and their families. Based on HCPs response, the PM Program was potentially less visible to HCPs than the V Program was. This could have been due to the time of day and frequency of offered interventions for each program (i.e., V interventions were offered three times per week during the day; PM interventions were mostly offered in the evenings and only scheduled once per week, though there was opportunity to connect with families at other pre-determined times).

An important theme that emerged from the HCP data is that in both pre- and post-data, PICU HCPs reported that they lack both time and training to provide regular developmental care to patients. This finding may be corroborated by answers in the V survey that indicated families did not feel that the PICU regularly offers age-appropriate activities to patients. However, as per Figure 4, it is possible that exposure to the P/V Program has helped to decrease the perception of barriers to developmental care in the PICU. It is also possible that exposure to the program and modeling by PMs helped HCPs to feel that it is within their job description to provide emotional support and guidance to families (Figure 5). These findings are important to PICU's operational leadership as they work to clarify expectations within their multidisciplinary team, and increase the skill and capacity of their HCPs to be able to offer developmental care to patients and support families at the bedside. This evaluation also illuminated the need to clarify roles and intervention scope between the PMs, Vs and the wider multidisciplinary healthcare team to ensure there remains a perception by all that the P/V Program is value-added to the PICU, and not redundant to the Social Worker and Child Life roles, for example.

Another interesting finding was that families reported two extremes of their availability to be physically present at the bedside with their child. We hypothesize that those families spending 8 h weekly in the PICU would benefit from this program in having trained Vs at their child's bedside to support non-medical interaction. Those families spending 168 h weekly (24/7), could benefit from the program in potentially taking self-care breaks away from bedside or receiving support from a PM. Fourteen families did not respond to this question, and we can only speculate if the lack of response was due to either their inconsistent presence in the PICU or guilt over not being as present as they would have liked. Further evaluation with these groups would be helpful to better understand eithers' experience. Overall, this program evaluation is an important step to identifying both areas for improvement and encouraging the work toward on-going P/V Program success and sustainability.

Prior to initiating our P/V Program, we conducted a snowball sampling environmental scan to determine how others had designed, developed and implemented a like program in other institutions. We found nine sites across North America that advertised a type of V program for their neonatal intensive care unit (NICU) or PICU. The majority of NICU programs employed Vs as “cuddlers” for their patient populations. PICU Vs were trained to offer developmentally appropriate non-medical interaction and play to patients, or to help families with way-finding.

In general, V programs in Canada and North America are thought to contribute to significant cost savings to hospitals and are also likely to enhance quality indicators such as patient satisfaction and safety (11). If reported, measure of effect was often reduced to a cost-benefit analysis, with cost attributed to approximate equivalent work-time of a paid employee (11). The literature is lacking in terms of information on the effect of a P/V program like ours on patient, family and HCPs experience in other pediatric hospitals.

### Limitations

This program evaluation has potential limitations. Surveys are limited by sampling bias whereby the results may not accurately reflect the experiences of those who did not respond. Survey data is also limited by the completeness of survey responses as respondents may have skipped questions or offered limited explanation for their selections. This limitation was partially addressed by triangulating data and reporting the “*n*” value where appropriate. Also of note, the results of our post-implementation surveys for families may have been influenced by the amount of time they spent on the unit. If families were not present to witness V services or engage with PMs, they may have had an incomplete perspective of the programs. In the post-implementation volunteer survey, we did not ask for the time families spent on the unit, so we were unable to correlate if their presence had an influence on their perspective of the programs. We would include this demographic data in subsequent evaluation questions. Finally, as we mentioned above, in March 2020, post-implementation evaluation data collection was halted due to the COVID-19 pandemic. Frontline HCPs were deployed to other units to address pandemic needs, families were limited to one caregiver at a time at their child's bedside, and Vs were not allowed on-site. Therefore, post-implementation survey responses were understandably lower across all participant groups. The results from all post-implementation surveys are limited by the small number of surveys we were able to collect compared to pre-implementation. We look forward to continuing this robust evaluation when COVID-19 restrictions are lifted at our hospital.

## Conclusion

The PICU P/V Program was designed to improve the experience of patients, families, and HCPs in the PICU. For patients, the opportunity to receive non-medical interaction has the potential to facilitate important growth and development and reduce the risk for PICS. For families, PMs may help them to more easily navigate their hospital experience as well as feel a type of comradery-support that they currently do not receive from medical professionals. For HCPs, the P/V Program may help to offset developmental care requirements so that they may focus on other quality of care activities.

In summary, the PICU's P/V Program Pilot was a success, offering potential benefit to patients and families and perceived value from our HCPs. We aim to continue this program once the COVID-19 restrictions on those families and volunteers allowed in the hospital are lifted. When we are able to re-initiate the program, we will focus on fostering awareness of the program for patients, families, and HCPs, and developing more clarity between our new support roles (PMs and Vs) and our existing support roles (Child Life Specialists and Social Work). There was also evidence that our HCPs could benefit from more focused developmental care education and training so they can better understand the hands-on, non-medical interaction that can be offered to acutely ill patients in the PICU.

This program evaluation is an important contribution to the literature in the field. There exists a paucity of empirical research dedicated to understanding the short- and long-term effects of PMs and Vs on patients, families, and HCPs in PICU settings. Further research is required to test the short-term effects of a similar P/V program on patients, families, and HCPs in in this type of environment. Longitudinal studies are required to elucidate the influence of PMs and Vs on PICS for patients and their families.

## Data Availability Statement

The original contributions presented in the study are included in the article/supplementary material, further inquiries can be directed to the corresponding author.

## Ethics Statement

The studies involving human participants were reviewed and approved by University of Alberta Research Ethics Board (Study ID Pro00089760). Written informed consent for participation was not required for this study in accordance with the national legislation and the institutional requirements.

## Author Contributions

NP, AD, CM, and AB composed and edited the manuscript. DG reviewed and edited the draft. All authors were involved in conceptualizing, developing, executing, and evaluating this project.

## Funding

We wish to acknowledge the *Alberta Health Services Chief Medical Office* for funding this quality improvement project.

## Conflict of Interest

The authors declare that the research was conducted in the absence of any commercial or financial relationships that could be construed as a potential conflict of interest.

## Publisher's Note

All claims expressed in this article are solely those of the authors and do not necessarily represent those of their affiliated organizations, or those of the publisher, the editors and the reviewers. Any product that may be evaluated in this article, or claim that may be made by its manufacturer, is not guaranteed or endorsed by the publisher.

## References

[1] Manning JC Pinto NP Rennick JE Colville G Curley MAQ. Conceptualizing post intensive care syndrome in children-the PICS-p framework. Pediatr Crit Care Med. (2018) 19:298–300. 10.1097/PCC.0000000000001476 29406379

[2] Watson RS Choong K Colville G Crow S Dervan LA Hopkins RO. Life after critical illness in children—toward an understanding of pediatric post-intensive care syndrome. J Pediatr. (2018) 198:16–24. 10.1016/j.jpeds.2017.12.084 29728304

[3] McPeake J Hirshberg EL Christie LM Drumright K Haines K Hough CL. Models of peer support to remediate post-intensive care syndrome: a report developed by the society of critical care medicine thrive international peer support collaborative. Crit Care Med. (2019) 47:e21–7. 30422863 10.1097/CCM.0000000000003497 PMC6719778

[4] Moody C Callahan TJ Aldrich H Gance-Cleveland B Sables-Baus S. Early initiation of Newborn Individualized Developmental Care and Assessment Program (NIDCAP) reduces length of stay: a quality improvement project. J Pediatr Nurs. (2017) 32:59–63. 10.1016/j.pedn.2016.11.001 27923536

[5] Montirosso R Giusti L Del Prete A Zanini R Bellu R Borgatti R. Does quality of developmental care in NICUs affect health-related quality of life in 5-y-old children born preterm? Pediatr Res. (2016) 80:824–8. 10.1038/pr.2016.158 27490739

[6] Pineda R Bender J Hall B Shabosky L Annecca A Smith J. Families participation in the neonatal intensive care unit: predictors and relationships to neurobehavior and developmental outcomes. Early Hum Dev. (2018) 117:32–8. 10.1016/j.earlhumdev.2017.12.008 29275070 PMC5856604

[7] Johnson BH Abraham MR. Partnering with Patients, Residents, and Families: A Resource for Leaders of Hospitals, Ambulatory Care Settings, and Long-Term Care Communities. Bethesda, MD: Institute for Patient- and Family-Centered Care. (2012).

[8] Meert KL Clark J Eggly S. Family-centered care in the pediatric intensive care unit. Pediatr Clin North Am. (2013) 60:761–72. 10.1016/j.pcl.2013.02.011 23639667 PMC3767974

[9] Just AC. Parent participation in care: bridging the gap in the pediatric ICU. Newborn infant Nurs Rev. (2005) 5:179–87. 10.1053/j.nainr.2005.08.002

[10] Wodinski LM Mattson McCrady HM Oswald CM Lyste NJM Forbes KLL. Family bedside orientations: an innovative peer support model to enhance a culture of family-centred care at the Stollery Children's Hospital. Paed Child Healt-Can. (2017) 22:387–90. 10.1093/pch/pxx117 29479254 PMC5804973

[11] Hotchkiss RB Unruh L Fottler MD. The role, measurement, and impact of volunteerism in hospitals. Nonprof Volunt Sec Q. (2014) 43:1111–28. 10.1177/0899764014549057

